# Transition Ramp Graft: A Simple and Effective Solution for Transitional Concavity Between the Nasal Dorsum and Tip Projection Grafts

**DOI:** 10.1007/s00266-026-05759-6

**Published:** 2026-03-17

**Authors:** Şeyda Güray, Erdem Tezel

**Affiliations:** Department of Plastic, Reconstructive and Aesthetic Surgery, Marmara University Pendik Training and Research Hospital, Istanbul, Türkiye

**Keywords:** Rhinoplasty, Supratip deformity, Cartilage grafting, Cleft nasal deformity, Revision rhinoplasty, Nasal contour

## Abstract

**Background:**

Contour irregularities at the dorsum–tip junction remain a challenge in primary, secondary, and cleft-related rhinoplasty, particularly in patients with scarring or shortened soft tissues. While this transition irregularity can often be reduced by lowering the dorsum in standard cases, it becomes problematic in heavily scarred patients who require long septal extension grafts to achieve adequate tip projection, thereby increasing the risk of an abrupt and aesthetically unfavorable dorsum–tip transition. To address this issue, we evaluated the Transition Ramp Graft, a small structural onlay designed to equalize the height between the dorsum and tip and improve contour harmony in anatomically complex cases.

**Methods:**

A retrospective review was conducted on patients who underwent open structural rhinoplasty with placement of the Transition Ramp Graft (TRG). Surgeon-based evaluations and patient-reported outcomes were assessed at 12 months.

**Results:**

The TRG was applied in 56 patients. Surgeons reported excellent contour continuity, and patient satisfaction was high. No graft-related complications were observed.

**Conclusions:**

The TRG provides a simple and reliable method for correcting dorsum–tip height discrepancies and improving contour harmony.

**Level of Evidence IV:**

This journal requires that authors assign a level of evidence to each article. For a full description of these Evidence-Based Medicine ratings, please refer to the Table of Contents or the online Instructions to Authors www.springer.com/00266.

## Introduction

In rhinoplasty, the presence of smooth, continuous, and aesthetically harmonious dorsal lines, together with a well-defined dorsal contour, represents one of the most critical determinants of postoperative satisfaction [1]. The transition between the nasal dorsum and the tip complex gains particular aesthetic significance when evaluated on the lateral profile, where even subtle contour irregularities become more apparent. [2]

In most patients, the transition between the dorsum and the tip can be achieved without creating a distinct deformity; however, in cases requiring substantial tip projection such as those with post-traumatic nasal deformities, cleft lip nasal deformities associated with columellar shortening or scarring or in patients with thick skin placement of additional tip grafts may be necessary to obtain sufficient structural support and tip projection. In such situations, a distinct transition zone tends to form between the dorsum and the tip. [3]

This zone may interfere with the natural formation of the supratip breakpoint and instead lead to an abrupt and more caudally positioned transition, resulting in a contour that appears aesthetically unbalanced and disharmonious with the overall nasal profile. In recent years, septal extension grafts have been increasingly utilized to provide long-term nasal tip support; however, their use may contribute to the development of this type of deformity. This tendency is particularly observed in certain technical modifications of the procedure, especially when the graft is positioned at a right angle (90 degrees) to the septum. In such configurations, the angular relationship between the graft and the septum predisposes the nasal contour to an unnatural transition at the supratip region, thereby increasing the likelihood of postoperative contour irregularities. [4]

While several techniques such as camouflage grafts, spreader grafts, diced cartilage, and dorsal preservation methods aim to manage contour irregularities, they mainly focus on the radix and dorsum. Only a limited number of studies specifically address the narrow but crucial transition zone between the dorsum and the augmented tip. [5, 6]

To resolve this issue, we developed the Transition Ramp Graft (TRG), a small onlay graft placed precisely at the dorsum tip interface. Rather than supporting projection, the TRG equalizes contour, smoothing height differences between the dorsum and tip grafts. It lifts the overlying skin to prevent shadowing and creates a more harmonious profile, especially in patients with thin skin or pronounced nasal tip.

## Materials and Methods

A retrospective cohort study was conducted, including all patients who underwent open primary rhinoplasty performed by the author between 2019 and 2023, following approval from the institutional review board. Patients who underwent placement of a TRG to correct contour irregularities at the dorsum–tip junction were identified. Among these, those who had primary, secondary, or cleft-related rhinoplasty procedures were included in the study cohort. Cases in which additional dorsal camouflage grafts were used in conjunction with the TRG were excluded from the analysis to ensure homogeneity of the study group.

Postoperative outcomes were evaluated at the 12 month follow-up using both surgeon-based and patient-reported measures. The smoothness and continuity of the dorsum tip contour were independently assessed by three experienced plastic surgeons, each using a five-point Likert scale (1 = poor, 5 = excellent) on standardized right and left lateral and frontal view photographs obtained at 12 months. Interrater reliability among observers was analyzed using Fleiss’ kappa statistics, which indicated a high level of agreement. Patient satisfaction with nasal contour and overall aesthetic outcome was assessed using a ten-point Visual Analog Scale (VAS) (0 = completely dissatisfied, 10 = completely satisfied). Complications such as infection, extrusion, or graft displacement were recorded through clinical examination and photographic documentation during follow-up visits.

## Surgical Technique

**Fig. 1:**
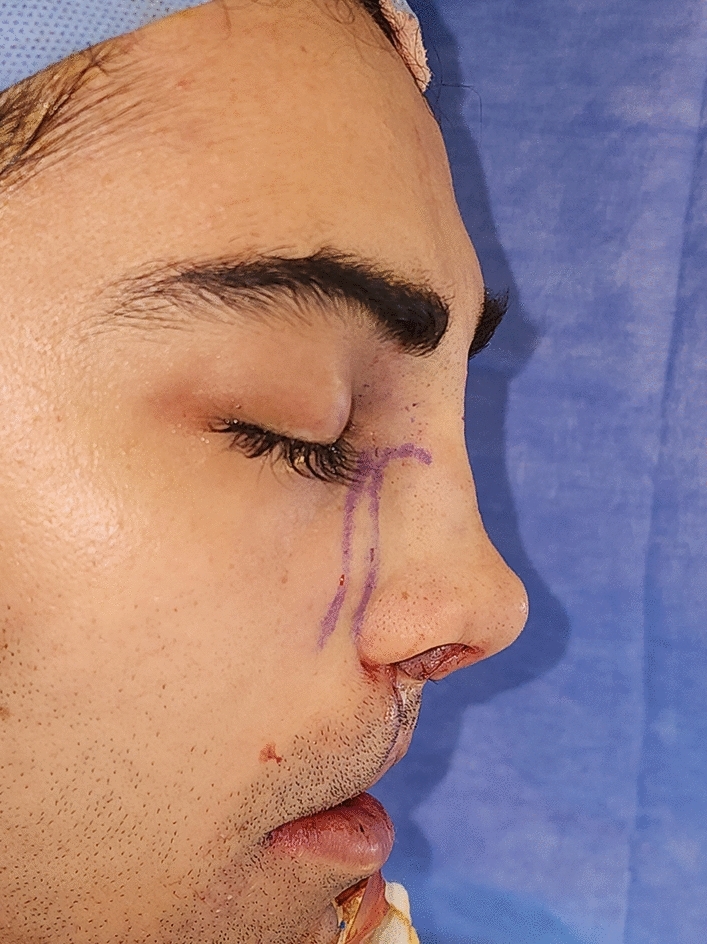
Intraoperative appearance of a patient who underwent septal extension grafting. Despite appropriate placement of the SEG, a distinct supratip break deformity is evident, indicating a collapse in the transition zone between the dorsum and the tip

**Fig. 2:**
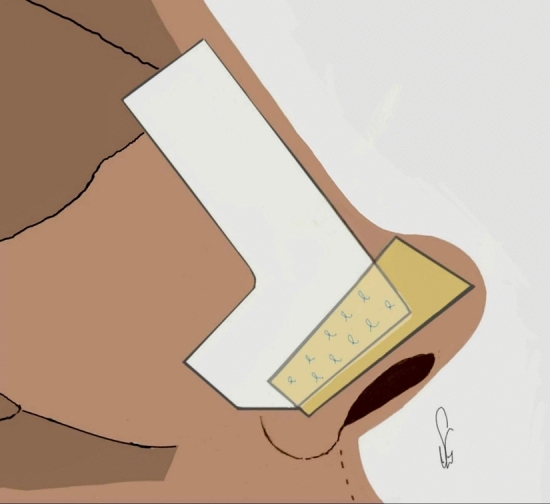
Established supratip break deformity following tip augmentation**.** This illustration demonstrates a distinct supratip break deformity that has developed after nasal tip augmentation. Despite adequate structural support with a septal extension graft, the failure to adequately blend the tip–dorsum junction has resulted in a pronounced step-off, disrupting dorsal aesthetic continuity

**Fig. 3:**
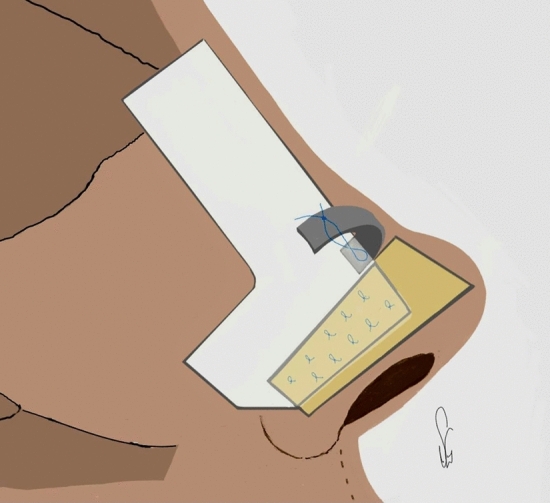
Placement of the Transitional Ramp Graft to smooth the tip–dorsum transition. This diagram illustrates the strategic positioning of the Transitional Ramp Graft at the junction of the nasal tip and dorsum. The graft functions as a three-dimensional spacer, designed to harmonize the sagittal and coronal contour planes. Its placement minimizes abrupt step-offs and reinforces the subcutaneous space to prevent supratip depression, particularly in patients with thin skin and rigid tip support structures

After all tip enhancing grafts were secured, the transition zone was evaluated. By “transition mismatch,” we refer to cases in which the geometric relationship between the nasal dorsum and the supratip region is discordant, resulting in an abrupt or misplaced transition point (Fig. 1). In such situations, neither excessive dorsal reduction nor tip deprojection alone provides a satisfactory solution, as these maneuvers may compromise dorsal aesthetic lines or long-term tip support. This mismatch is often accompanied by a misplaced supratip breakpoint, contributing to visible discontinuity between the dorsum and the nasal tip. If there are no indications requiring graft placement along the entire nasal dorsum such as inadequate dorsal projection, irregularities, or deformities along osteotomy lines, the presence of an isolated deficiency confined to the supratip break point represents the primary indication for the use of this cartilage graft (Figs. 2, 3). In cases with visible supratip depression often due to thin skin or high projection, an autologous cartilage graft was harvested (from septum, concha, or rib) and shaped into a thin, rectangular-shaped onlay.

**Fig. 4:**
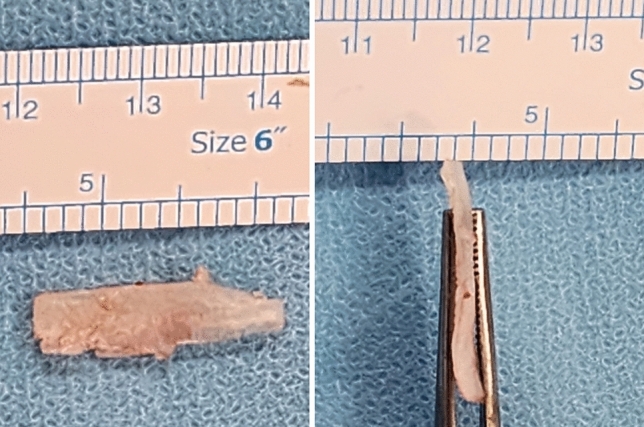
The cartilage block is tangentially thinned with an 11-blade scalpel to approximately 1 mm thickness, achieving the desired curvature and pliability. The final graft, measuring roughly 20 mm in length and 5 mm in height, demonstrates the appropriate configuration for supporting the dorsum tip transition

The septal cartilage serves as the primary donor source for graft preparation. In cases where the septal cartilage is insufficient or unavailable, conchal or costal cartilage may be utilized as alternative sources. Owing to the inherent rigidity of costal cartilage, meticulous thinning is required to achieve an appropriate saddle-shaped configuration. A tangentially shaped graft, made with an 11-blade scalpel, measuring approximately 1 mm in thickness, 20 mm in width, and 5 mm in height, provides an optimal form for this purpose (Fig. 4).

**Fig. 5:**
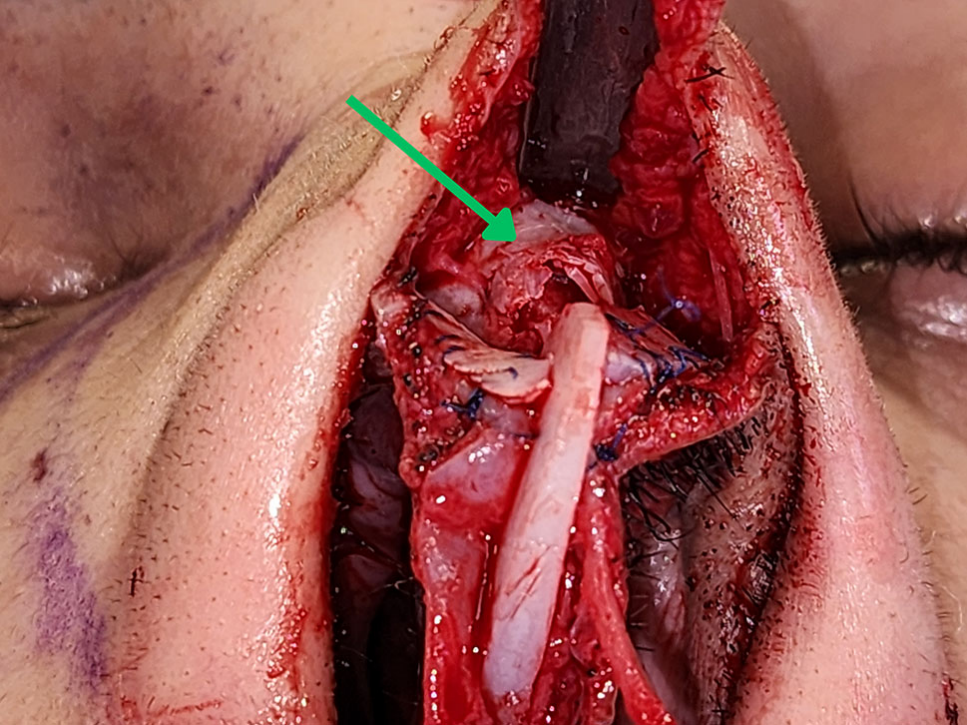
Intraoperative view demonstrating the placement of grafts. The vertically positioned graft in the foreground is the septal extension graft, providing anterior tip support. Transitional Ramp Graft (TRG) (green arrow) is designed to smooth the dorsal–tip transition and prevent supratip depression

**Fig. 6:**
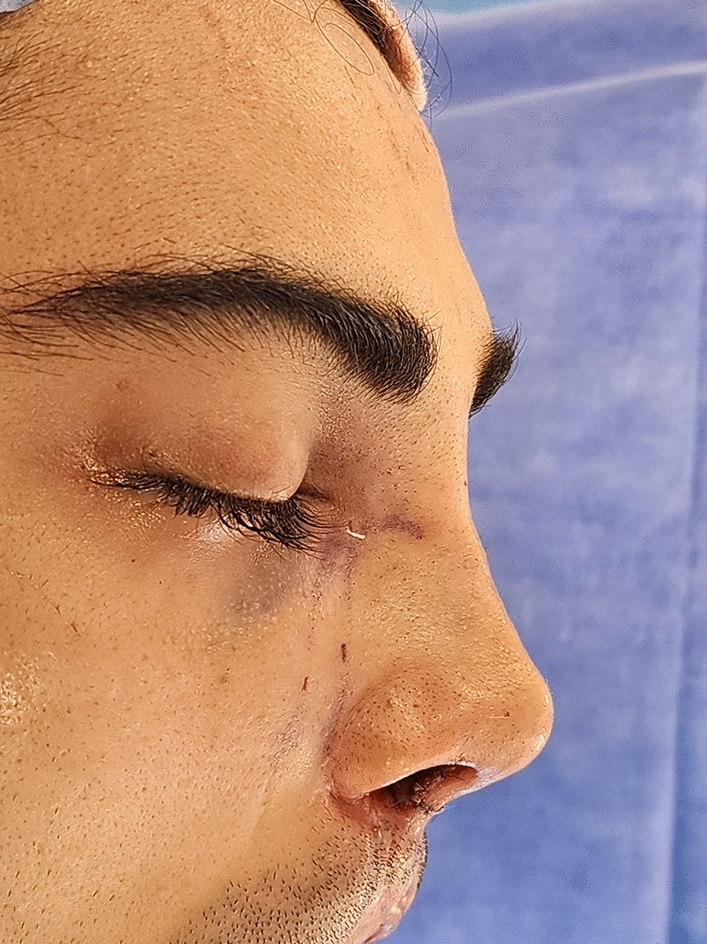
Intraoperative image of the same patient after placement of the Transitional Ramp Graft (TRG). The graft effectively smooths the transition between the dorsum and the tip, eliminating the previously observed supratip break. The nasal profile shows a more continuous and stable contour, demonstrating the graft’s role in restoring aesthetic harmony in the transition zone

**Fig. 7:**
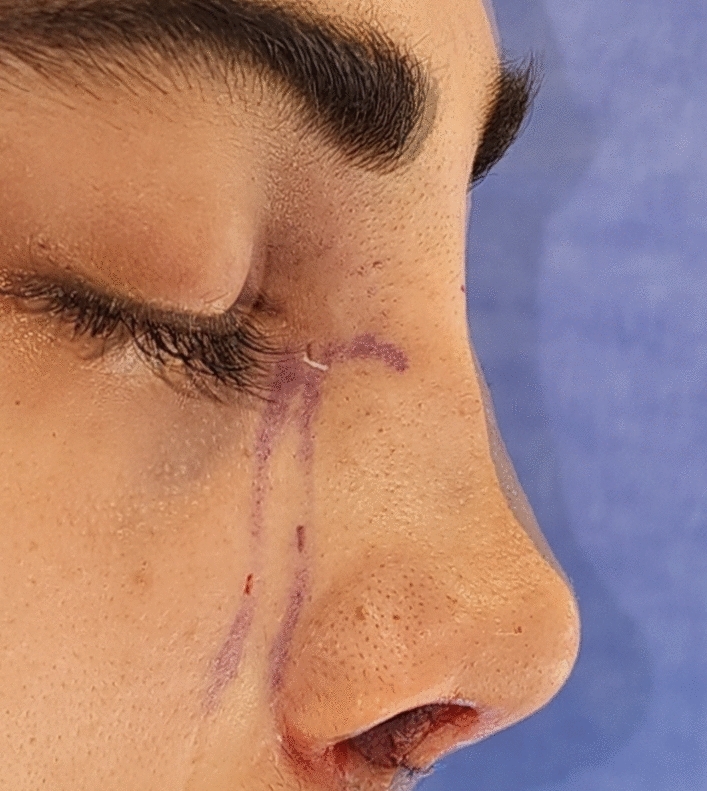
Superimposed intraoperative images showing the dorsal–tip contour before and after placement of the Transitional Ramp Graft. The supratip break observed in the pre-graft profile is effectively eliminated following graft placement, resulting in a smoother and more continuous dorsal aesthetic line

The graft was contoured to match the sagittal and coronal planes of the depression and anchored to the septum using 5-0 polypropylene sutures (Fig. 5). A non-absorbable suture was passed through one limb of the graft, the septum, and the opposite limb, then looped back in a horizontal mattress, and tied over cartilage. The intentional subgraft space allows the TRG to behave as a controlled, spring like support system (Figs. 6, 7). Rather than relying on tissue contraction or fibrosis, the graft functions as a preformed architectural platform that gently lifts and stabilizes the overlying skin envelope. The inherent curvature and elastic behavior of the graft maintain a consistent separation between the skin and the underlying framework, preventing supratip adhesion and ensuring a smooth transition during healing.

## Results

**Fig. 8:**
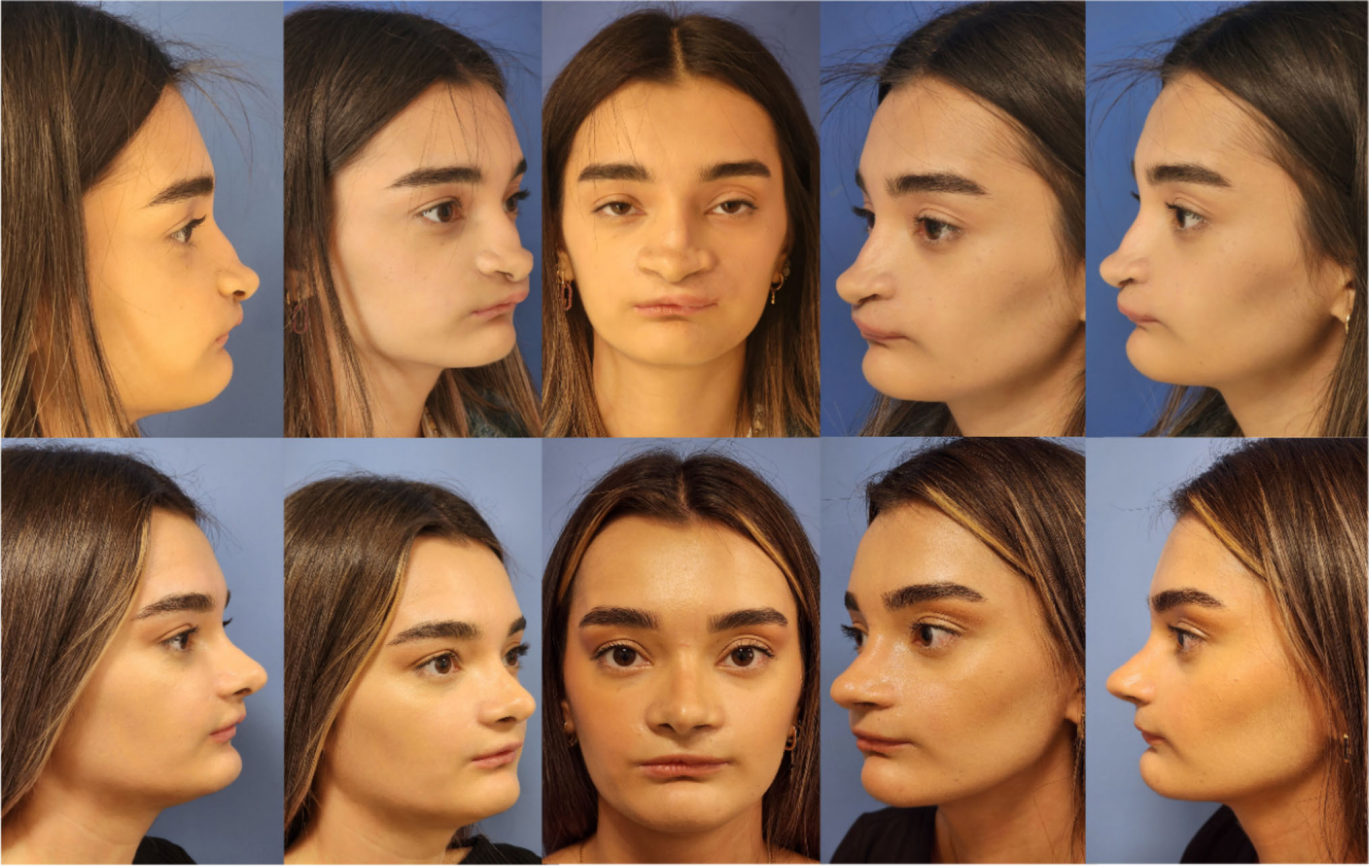
The upper row photographs, from left to right, demonstrate the patient’s preoperative appearance after maxillary distraction, showing marked nasal deformity, insufficient tip support, and a dorsum requiring augmentation in the left lateral, left oblique, anterior, right oblique, and right lateral views. The lower row photographs, from left to right, show the corresponding one-year postoperative left lateral, left oblique, anterior, right oblique, and right lateral views

**Fig. 9:**
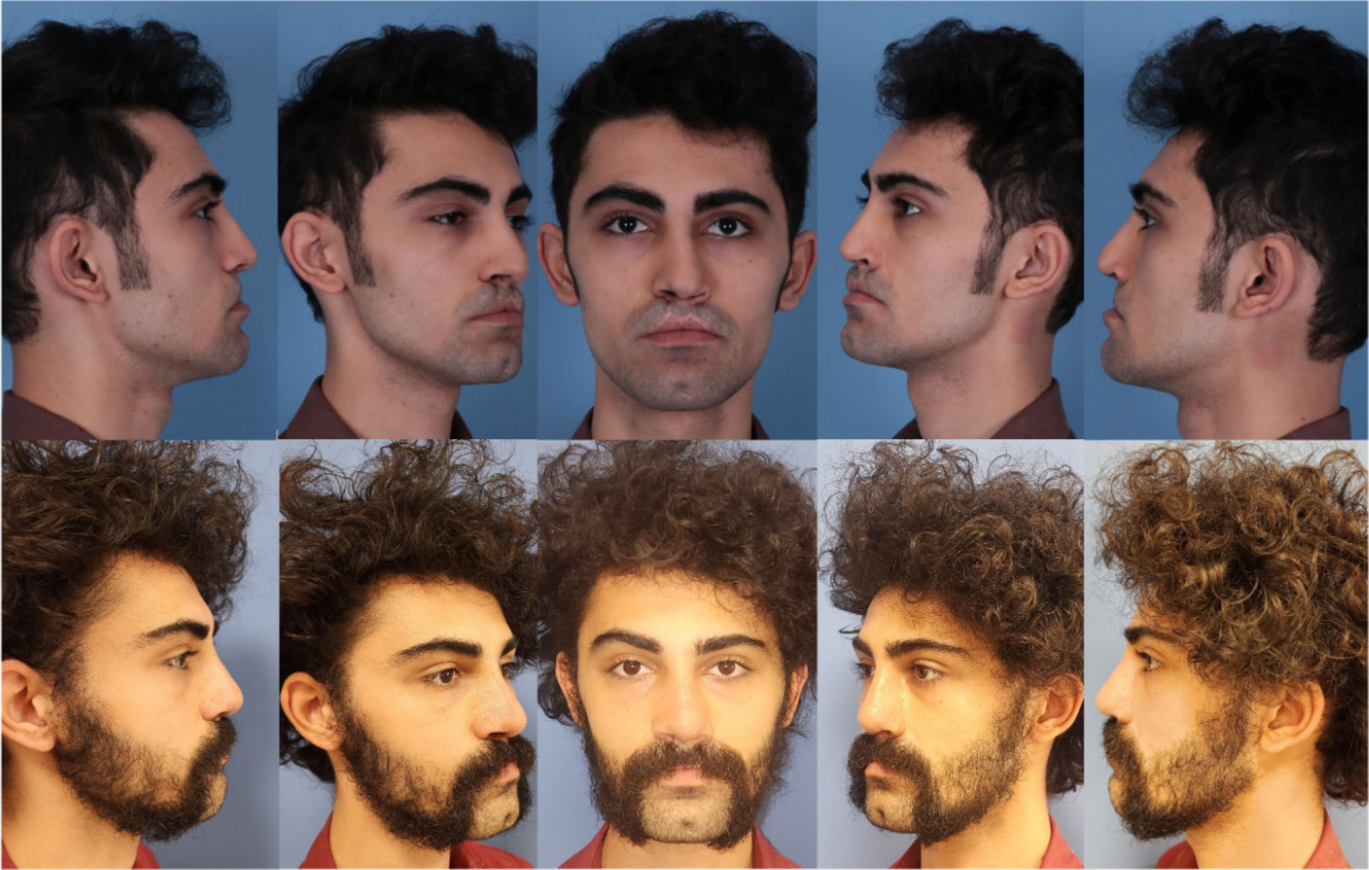
The upper row photographs, from left to right, demonstrate the patient’s preoperative appearance of a unilateral cleft lip nasal deformity in the left lateral, left oblique, anterior, right oblique, and right lateral views. The lower row photographs, from left to right, show the postoperative results at one-year follow-up in the corresponding views. This patient is the same case presented with the intraoperative views illustrating the application of the Transitional Ramp Graft. The transition achieved between the nasal tip and dorsum during surgery is maintained in a stable and harmonious manner at the one-year follow-up

At the 12-month follow-up, postoperative outcomes were evaluated using standardized lateral and frontal photographs. Surgeon-based assessments using a five-point Likert scale demonstrated excellent contour smoothness. Lateral view evaluations yielded a mean score of 4.6 ± 0.5, whereas frontal view assessments showed slightly lower scores, with a mean of 4.3 ± 0.6. This difference is consistent with the greater sensitivity of the frontal view to minor asymmetries and midline deviations. Interrater reliability was high for both assessment planes, with Fleiss’ kappa values of 0.82 for lateral evaluations and 0.78 for frontal evaluations, indicating substantial agreement among observers (Figs. 8, 9).

Patient-reported outcomes demonstrated high overall satisfaction, with a mean Visual Analog Scale score of 8.9 ± 1.1. No complications such as infection, graft extrusion, or displacement were observed throughout the follow-up period. The most pronounced aesthetic improvements were observed in patients undergoing secondary rhinoplasty and in those with cleft-associated nasal deformities, in whom dorsum–tip transition irregularities are typically more severe and challenging to correct.

## Discussion

The need for a more refined tip definition becomes particularly relevant in patients with cleft lip nasal deformities and in those undergoing secondary or tertiary rhinoplasty, where extensive scarring and soft tissue contracture around the columella and nasal tip further compromise projection and mobility. In this combined patient group, if the dorsum is also under-projected, excessive dorsal reduction may further worsen the mismatch between the dorsum and the tip. Furthermore, the presence of shortened and scarred soft tissues frequently necessitates the use of a long septal extension graft to achieve adequate tip support and projection. For these reasons, minimizing dorsal reduction while maximizing tip definition is essential an interplay that makes the dorsum tip transition particularly vulnerable to contour irregularities. Although secondary healing and fibrosis can partially compensate for this gap, the outcome remains unpredictable and aesthetically insufficient. Consequently, targeted and reproducible structural interventions are essential to restore a smooth and natural transition between the dorsum and the nasal tip. The TRG is especially advantageous in such cases because it compensates for limited dorsal projection and provides a smoother, more harmonious transition despite the need for strong tip support.

Various techniques have previously been described to address contour irregularities in the supratip and dorsal regions. Lyrio et al. introduced the supratip cross flap, a cartilage-based flap derived from the cephalic portion of the lower lateral cartilage, crossed over the midline to reinforce the supratip and external valve region. [7] The technique offers contour improvement and airway support but depends on the integrity and mobility of the lower lateral cartilages, which are often compromised in secondary or cleft cases

Öztürk proposed preserving the Pitanguy ligament to enhance supratip definition and to help accurately position the supratip breakpoint without creating an undesirably contour [8]. In his approach, the ligament is dissected but left intact, and its midportion is sutured to a premarked septal point, thereby functioning as a suspensory structure that maintains supratip contour stability. While this technique allows refined tip definition in thin to moderately thick skinned patients, it relies primarily on soft tissue dynamics rather than structural modification, which limits its predictability in revision or cleft-related deformities.

Beyond these soft tissue or flap-based strategies, recent literature has emphasized cartilage derived onlay grafts for smoothing surface irregularities in the dorsum-supratip complex. Lee and Park introduced the septal gap graft as an autologous method to correct supratip depression following tip projection without implants, primarily in Asian patients undergoing primary rhinoplasty. [9] Their technique addresses the vertical height discrepancy between the nasal dorsum and the elevated tip by interposing septal cartilage between the upper lateral cartilages and the tip complex. Although satisfactory results have been reported in selected primary cases with sufficient septal cartilage, its applicability is limited by septal cartilage availability, reliance on intact lateral cartilage anatomy, and the need for rigid fixation between the upper and lower lateral cartilages, which may reduce physiologic tip mobility, particularly in high-projection or revision cases.

In contrast, the Transition Ramp Graft was designed to treat the dorsum–tip transition as a distinct anatomical subunit rather than simply filling a vertical gap. Acting as a thin, structurally contoured onlay graft bridging the dorsum and tip projection grafts, the TRG provides controlled mechanical support to the overlying skin envelope without tethering the nasal tip to the dorsal framework. Unlike the septal gap graft, which is largely restricted to primary rhinoplasty, the TRG can be harvested from septal, conchal, or costal cartilage and remains effective in anatomically challenging environments. Furthermore, whereas the septal gap graft primarily restores sagittal continuity, the TRG improves both sagittal and coronal contour planes by maintaining subcutaneous space and preventing supratip adhesion during healing, resulting in more consistent and stable outcomes without reliance on diced cartilage or fascia-wrapped grafts. This mechanism is also reflected in our evaluation outcomes. The difference between lateral and frontal scores observed in the Likert scale assessment indicates that the graft technique achieved its intended objective. While the frontal view is useful for assessing asymmetries, dorsal harmony, and angular relationships such as the nasolabial, nasofrontal, and lobular angles, the lateral view is more informative for evaluating the transition between the nasal tip and the dorsum. From this perspective, when asymmetry is set aside, obtaining more favorable results in the lateral view for assessing improvement in the transitional zone is not an unexpected finding.

Boccieri et al. demonstrated that moderately crushed cartilage achieves the optimal balance between flexibility and stability, whereas excessive fragmentation increases resorption and recurrence of supratip deformities. [10]

Ledo et al. further reported that free diced cartilage grafts used in more than 4000 rhinoplasties yielded extremely low complication and revision rates resorption in only 0.67% and reoperation in 1.5% of cases highlighting their reliability as camouflage material [11]. In parallel, Keyhan et al. found that diced cartilage wrapped in fascia produced similarly low complication rates, with pooled resorption, infection, and revision rates of 2.5%, 2.3%, and 3.0%, respectively. [12]

Collectively, these techniques serve primarily as passive fillers that mask contour irregularities and enhance surface smoothness. However, they do not actively restore the anatomical and mechanical continuity between nasal subunits. Moreover, diced or crushed cartilage grafts can undergo variable resorption and depend on surrounding soft tissue support, whereas fascia-wrapped grafts add procedural complexity and require additional donor sites.

In contrast, TRG offers an active, mechanically supported, and biologically adaptive solution. Rather than passively camouflaging depressions, the TRG reestablishes dorsum tip continuity through a three-dimensional structural bridge. Furthermore, the TRG eliminates the need for fascia wrapping or donor site harvesting, reducing operative time and morbidity. Its independence from lower lateral cartilage integrity also permits consistent application in thin-skinned, cleft-related, or post-traumatic deformities where flap-based or filler approaches may fail. Extending this principle to the dorsum tip interface, the TRG provides continuous support that maintains harmony during postoperative healing. In the sagittal plane, it smooths height discrepancies between the dorsum and tip; in the coronal plane, it lifts and supports the skin envelope against the restrictive effects of fibrotic adhesions and shortened soft tissues

Although primarily developed for the treatment of internal nasal valve insufficiency, the butterfly graft has also been shown to influence dorsal contour aesthetics due to its anatomical placement. By spanning the internal nasal valve apex, the graft may secondarily soften transition lines along the nasal dorsum and supratip region. Despite its well-documented functional benefits, the butterfly graft has also been associated with certain aesthetic concerns. Several authors have reported postoperative supratip fullness, increased supratip width, and occasional graft visibility, particularly in patients with thin skin or narrow midvault anatomy [13, 14]. These effects are largely attributable to the graft’s position at the internal nasal valve apex and its inherent tendency to increase structural bulk in the supratip region. Although technical refinements such as meticulous graft contouring, edge beveling, and more caudal positioning have significantly improved aesthetic outcomes, the potential for supratip widening remains an important consideration during patient selection and surgical planning., including butterfly grafts, onlay supratip, or dorsal camouflage grafts. Butterfly grafts, typically harvested from auricular cartilage and positioned over the internal nasal valve region, primarily aim to improve airway function while providing secondary contour smoothing effects. Although effective in selected cases, their indication is mainly functional, and their contribution to fine dorsal–tip contour modulation remains limited, particularly in patients requiring strong structural tip support or those with significant scarring. In contrast, the Transition Ramp Graft is designed as a true interface graft that provides a controlled structural transition between the nasal dorsum and the tip complex. Unlike butterfly grafts, it allows refinement of dorsal–tip continuity without increasing supratip bulk or width, making it particularly advantageous in patients requiring precise contour modulation and strong tip support.

## Conclusion

In summary, the Transition Ramp Graft offers a simple and reliable structural solution for correcting dorsum–tip height discrepancies, especially in cleft and revision rhinoplasty where scarring and limited dorsal projection challenge contour harmony. By providing a stable, supportive bridge that maintains the skin envelope during healing, the TRG achieves a smoother and more natural transition than soft tissue or camouflage-based methods, ensuring durable aesthetic continuity of the nasal profile.

## References

[1] Byrd HS, Andochick S, Copit S, Walton KG. Septal extension grafts: a method of controlling tip projection shape. Plast Reconstr Surg. 1997;100(4):999–1010. 9290671 10.1097/00006534-199709001-00026

[2] Dibelius G, Hohman MH. Rhinoplasty tip-shaping surgery. In: StatPearls. Treasure Island, FL: StatPearls Publishing. 2025. 33620827

[3] Daoud RM, Alelyani AA, Bakhamees BH, et al. Thin skin in rhinoplasty: considerations for camouflaging dorsal nasal irregularities. Cureus. 2024;16(8):e66595. 10.7759/cureus.66595. 39258084 10.7759/cureus.66595 PMC11383643

[4] Rohrich RJ, Durand PD, Dayan E. Changing role of septal extension versus columellar grafts in modern rhinoplasty. Plast Reconstr Surg. 2020;145(5):927e-e931. 10.1097/PRS.0000000000006730. 32332531 10.1097/PRS.0000000000006730

[5] Santos M, Ribeiro A, Almeida ESC, et al. Shaved cartilage gel versus diced cartilage on final dorsal camouflage: prospective study of 200 patients. Fac Plast Surg Aesthet Med May-Jun. 2021;23(3):164–71. 10.1089/fpsam.2020.0180. 10.1089/fpsam.2020.0180 32721239

[6] Hanci D, Ustun O, Kumral TL, Ahmed EA, Uyar Y. Camouflage of the nasal dorsum in thin-skinned patients with diced cartilage combined with a new cross-linked hyaluronan (NCH) gel and blood: a new method. Aesthet Plast Surg. 2019;43(3):786–92. 10.1007/s00266-019-01323-7. 10.1007/s00266-019-01323-7 30783722

[7] Lyrio TM, Ferreira MG, Torres PG, Santos M, Tamanqueira P. Supratip cross flap: reshaping the nasal tip and improving the nasal vault function. Fac Plast Surg Aesthet Med. 2025;27(3):271–4. 10.1089/fpsam.2024.0050. 10.1089/fpsam.2024.0050 38708628

[8] Ozturk G. Controlling supratip break and tip rotation and projection with preservation of the Pitanguy Ligament. Plast Reconstr Surg. 2023;152(5):1002–6. 10.1097/PRS.0000000000010468. 36988451 10.1097/PRS.0000000000010468

[9] Lee W, Park B. A novel technique for autologous supratip augmentation: septal gap graft. J Craniofac Surg. 2022;33(4):1209–13. 10.1097/SCS.0000000000008278. 34711758 10.1097/SCS.0000000000008278

[10] Boccieri A, Marianetti TM, Pascali M. Crushed cartilage: a rescue procedure in rhinoplasty. J Craniofac Surg. 2018;29(3):614–7. 10.1097/SCS.0000000000004333. 29461374 10.1097/SCS.0000000000004333

[11] Ledo TO, Ramos HHA, Buba CM, et al. Outcome of free diced cartilage grafts in rhinoplasty: a systematic review. Facial Plast Surg. 2021;37(1):117–21. 10.1055/s-0040-1714664. 32886948 10.1055/s-0040-1714664

[12] Keyhan SO, Ramezanzade S, Bohluli B, Fallahi HR, Shakiba M, Yates J. A systematic review and meta-analysis of complications associated with autogenous diced cartilage wrapped in fascia used in nasal dorsum augmentation. Aesthet Surg J. 2021;41(9):Np1152–65. 10.1093/asj/sjab117. 34387329 10.1093/asj/sjab117

[13] Loyo M, Gerecci D, Mace JC, Barnes M, Liao S, Wang TD. Modifications to the butterfly graft used to treat nasal obstruction and assessment of visibility. JAMA Fac Plast Surg. 2016;18(6):436–40. 10.1001/jamafacial.2016.0681. 10.1001/jamafacial.2016.0681 27390095

[14] Rist TM, Clark JM. Indications and evolution of the butterfly graft in nasal valve repair. Otolaryngol Clin North Am. 2025;58(2):279–93. 10.1016/j.otc.2024.07.026. 39244465 10.1016/j.otc.2024.07.026

